# De Novo Mutations in Patients with Ataxic CP

**DOI:** 10.15844/pedneurbriefs-29-8-5

**Published:** 2015-08

**Authors:** Sonika Agarwal, Lisa Emrick

**Affiliations:** 1 Departments of Neurology and Developmental Neuroscience, Baylor College of Medicine, Houston, TX

**Keywords:** Cerebral Palsy, Ataxia, Genetics

## Abstract

As a part of a large study investigating childhood ataxias in the UK and Switzerland, Schnekenberg et al. analyzed the genetic associations with congenital cerebellar ataxia in 10 patients using either a targeted next generation sequencing panel of 118 genes or trio-based exome sequencing.

As a part of a large study investigating childhood ataxias in the UK and Switzerland, Schnekenberg et al. analyzed the genetic associations with congenital cerebellar ataxia in 10 patients using either a targeted next generation sequencing panel of 118 genes or trio-based exome sequencing [1]. The testing identified de novo mutations in three different genes, KCNC3, ITPR1 and SPTBN2 in 4 patients. Three of the four patients fulfilled criteria for ataxic cerebral palsy (lack of clinical or imaging regression and absence of syndromic features). The similarity in the phenotypes leads the authors to propose shared molecular mechanisms of pathogenicity. In three cases the combination of bioinformatics and electrophysiology combined with previous reports supported the variants to be pathogenic. In the 4th case, the novel variant was de novo, the authors classified it as a possible mutation. The fathers of these four cases all ranged from 34-40 years old. The identification of specific, proven pathogenic mutations leads the authors to suggest use of DNA sequencing for patients with ataxic CP [2, 3].

COMMENTARY. Cerebral palsy (CP) is a sporadic disorder with multifactorial etiologies, frequently associated with birth asphyxia, though a cause may not always be found. If no evidence of adverse perinatal events or imaging abnormalities are found, genetic work up is warranted. Ataxic CP is one of the least common types of CP and was thought to be inherited as an autosomal recessive trait in almost 50% of the cases in a study in 1992 [4]. However, recent WES data shows higher incidence of de novo mutations as cause of genetic disorders than expected [5]. The report found a de novo mutation or likely mutation in 4/10 patients in genes previously reported in familial ataxia syndromes, expanding the phenotypes for these genes.

The study uses good methodology using SNPs to confirm parentage and functional studies if available to classify variants as pathogenic in 3/4 patients. Trio-WES sequencing has higher yield in detecting de novo mutations than traditional WES, without compromising the diagnosis of recessive conditions. The study highlights the association of advanced paternal age with de novo mutations, thought at least to be due to DNA methylation abnormalities in the age-related sperm [2].

Determining a genetic etiology for a patient with CP is important for prognosis (static or progressive disorder), recurrence risk and possible treatments in the future.

The phenotype of ataxic CP may include hypotonia, tremors, seizures, auditory and speech impairment, and may be confused with other progressive genetic disorders. We would recommend a step wise approach to the diagnostic evaluation to include assessment of the clinical phenotype and imaging characteristics, and to look for the “red flags” [6], which may suggest the utility of DNA sequencing either next generation sequencing panel or trio-based whole exome sequencing.
